# Antioxidant Potential of Grass Pea Seeds from European Countries

**DOI:** 10.3390/foods7090142

**Published:** 2018-09-01

**Authors:** Wojciech Rybiński, Magdalena Karamać, Katarzyna Sulewska, Andreas Börner, Ryszard Amarowicz

**Affiliations:** 1 Institute of Plant Genetics, Polish Academy of Sciences, Strzeszyńska 34, 60-479 Poznań, Poland; wryb@igr.poznan.pl; 2 Institute of Animal Reproduction and Food Research, Polish Academy of Sciences, 10-748 Olsztyn, Poland; m.karamac@pan.olsztyn.pl (M.K.); k.sulewska@pan.olsztyn.pl (K.S.); 3 Leibniz Institute of Plant Genetics and Crop Plant Research, D-06466 Gatersleben, Germany; boerner@ipk-gatersleben.de

**Keywords:** grass pea, *Lathyrus sativus*, phenolic compounds, antioxidant activity

## Abstract

Phenolic compounds were extracted from seeds of 30 varieties of grass pea (*Lathyrus sativus*) into 80% (*v*/*v*) methanol. The total phenolics compounds content of the extracts and their antioxidant activity were determined using Folin-Ciocalteu’s phenol reagent and 2,2′-azinobis-(3-ethylbenzothiazoline-6-sulfonic acid) (ABTS) and ferric-reducing antioxidant power (FRAP) methods, respectively. Total phenolic contents ranged from 1.88 to 7.12 mg/g extract and 20.3 to 70.3 mg/100 g seeds. The extracts and seeds were characterized using Trolox equivalent antioxidant capacity values of 0.015–0.037 mmol Trolox/g extract and 0.158–0.372 mmol Trolox/100 g seeds, and FRAP values of 0.045–0.120 mmol Fe^2+^/g extract and 0.487–1.189 Fe^2+^/100 g seeds. The total phenolics content of grass pea extract was correlated with the results of the ABTS (*r* = 0.881) and FRAP (*r* = 0.781) assays. The same correlation was observed between the results of both assays (*r* = 0.842). Two derivatives of *p*-coumaric acid were the dominant phenolic compounds of the Derek cultivar of grass pea.

## 1. Introduction

Grass pea (*Lathyrus sativus*) is an ideal legume for resource-poor farmers, characterized by drought tolerance and thriving with minimal external inputs [1]. It is cultivated in the Indian subcontinent, Ethiopia, and to a lesser extent in North Africa, Australia, Asia, and Europe [2]. Currently, grass peas, similar to other legumes such as chickpea, lentil, and vetch are beginning to be cultivated in the Old World [3]. Grass pea seeds have a high nutritional value [4]. The protein, starch, lipids, mineral, and energy content in grass peas is similar to those of peas and faba beans [5]. For example, according to literature data, the protein content in grass pea, pea, and faba bean seeds is 26.5, 20.6, and 19–30 g/100 g, respectively [5,6,7]. The fatty acid profile of grass pea lipids is valuable. A high percentage of stearic acid was determined in grass pea lipids by Mehmet [8]. In epidemiologic and clinical studies, stearic acid was found to be associated with lowered low-density lipoprotein (LDL) cholesterol in comparison with other saturated fatty acids [9].

Unfortunately, grass pea seeds contain a neurotoxin, β-*N*-oxalyl-1-α,β-diamino-propionic acid (β-ODAP). This non-protein amino acid causes neurolathyrism, a neurological disease in humans and domestic animals [10]. The β-ODAP content of traditional grass pea cultivars is 0.5–2.5%. Genetic improvement of grass pea has reduced this content to <0.10% [11]. Soaking and boiling considerably reduces the content of β-ODAP in grass pea seeds [12,13]. Grass pea seeds can be used as a high-value protein source after protein extraction and the removal of antinutritional components [14]. Suitable functional properties (water absorption capacity, oil absorption capacity, foaming capacity, and foaming stability) of grass pea proteins were reported by Aletor et al. [15].

Legumes are a potentially valuable crop with high antioxidant potential [16]. The antioxidant and antiradical activities of leguminous seed extracts have been investigated using a variety of methods including liposomes, enhanced chemiluminescence, a β-carotene-linoleate model system, 2,2′-diphenyl-1-picrylhydrazyl (DPPH) and 2,2′-azinobis-(3-ethylbenzothiazoline-6-sulfonic acid) ABTS assays, the reducing power assay, LDL cholesterol oxidation, ferric-reducing antioxidant power (FRAP) assay, Fe^2+^-chelating capacity assay, and the hydrophilic oxygen radical absorbance capacity (ORAC_FL_) assay [17]. 

The reported content of total phenolics of grass pea flour was 0.22 and 0.27 g/100 g [18]. The phenolic content of grass pea extracts was correlated with their antioxidant properties determined using DPPH, FRAP, and β-carotene bleaching methods [19]. Menga et al. [20] reported linear correlations between the content of total phenolics, total flavonoids, and condensed tannins and results of the ABTS assay for grass pea extracts (*p* < 0.001). Total phenolic and condensed tannin levels were not correlated with seed yield and seed protein content in grass pea [21]. Grass peas extract inhibited α-amylase and α-glucosidase in an in vitro bioassay [19]. Results obtained by Stanisavljević et al. [22] strongly suggest that simple cooking treatment and in vitro digestion of grass pea seed flour applied prior to extraction with methanol could improve the antioxidative activity of the obtained extracts. 

The present study aimed to determine the total phenolic content of grass pea extracts and seeds as well their antioxidant activity and potential. To the best of our knowledge, this is the first publication to consider such a broad biological material from several countries.

## 2. Materials and Methods 

### 2.1. Plant Material

Plant material consisted of a collection of 30 grass pea varieties obtained in a field experiment conducted in Cerekwica (51°55′ N, 17°21′ E) derived from Italian, Spanish, French, German, and Polish lines. Descriptors for *Lathyrus sativus* were used (IPGRI 2000) for the evaluation and characterization of the phenotypic features of the new lines. The growth habit of each line was recorded at 50% flowering and scored as prostrate, spreading, semi-erect or erect. Flower colors were scored as blue, pink, red, white, or various combination of these colours. Pod shapes were scored as oblong, medium, oblong elliptical, curved, broad, broad-linear/elliptical, or a combination of these shapes. Seed coat color and shape were recorded on 100 randomly selected seeds immediately after threshing. Seed shape was generally classified as angled or wedge-shaped. After harvest, 10 randomly selected plants from each accession were chosen for estimation of quantitative traits (yield structure parameters). The weight of 100 seeds was calculated from weighing and counting at least 200 seeds. Until extraction, the seeds were stored in a refrigerator closed in vacuum bags. The characteristics of those seeds are reported in Table 1.

### 2.2. Chemicals

Sodium persulfate, ferrous chloride, Folin-Ciocalteau’s phenol reagent, 2,2′-azinobis-(3-ethylbenzothiazoline-6-sulfonic acid) (ABTS), 2,4,6-tri(2-pyridyl)-*s*-triazine (TPTZ), 6-hydroxy-2,5,7,8-tetramethyl-chroman-2-carboxylic acid (Trolox), and (+)-catechin were purchased from Sigma (Poznań, Poland). Acetonitrile high-performance liquid chromatography (HPLC) grade and methanol were obtained from P.O.Ch. Company (Gliwice, Poland).

### 2.3. Extraction

Phenolic compounds were extracted from ground seeds using 80% (*v*/*v*) methanol at a solids to solvent ratio of 1:10 (*w*/*v*) for 15 min at 50 °C [23]. The extraction was repeated twice, the supernatants were filtered and combined, and methanol was evaporated under vacuum in a R-200 rotary evaporator (Büchi Labortechnik AG, Flawil, Switzerland). The remaining aqueous solution was lyophilized.

### 2.4. Total Phenolic Compounds Content

The method described by Naczk and Shahidi [24] was used to determine the total phenolic compounds content of the extracts. Briefly, a 0.5-mL aliquot of seed extract dissolved in methanol was pipetted into a test tube containing 8 mL distilled water. After mixing the contents, 0.5 mL Folin-Ciocalteu’s phenol reagent and 1 mL saturated sodium carbonate solution were added. The contents were vortexed for 15 s and then left to stand at room temperature for 30 min. Absorbance measurements were recorded at 725 nm using a Beckman DU 7500 Spectrophotometer (Beckman Poland, Warsaw, Poland). Estimation of the phenolic compounds was carried out in triplicate. The results are expressed as (+)-catechin equivalents per g of the extract or 100 g seeds.

### 2.5. Condensed Tannins

Condensed tannins were determined using a vanillin/HCL colorimetric method [25]. The results obtained are reported as absorbance units at 500 nm per 1 mg extract.

### 2.6. ABTS Assay

The Trolox equivalent antioxidant capacity (TEAC) was determined using a method described by Re et al. [26]. Here, ABTS^+^ solution was prepared by mixing an ABTS stock solution in water with 2.45 mM sodium persulphate. This mixture was allowed to stand with shaking for 12–16 h at room temperature in the dark until reaching a stable oxidative state. For analysis, the ABTS^+^ stock solution was diluted with methanol to an absorbance of 0.720 at 734 nm. For the spectrophotometric assay, 2 mL ABTS^+^ reagent and 20 µL plant extract were mixed and the absorbance was read at 734 nm at 37 °C for 10 min. A calibration curve was plotted using Trolox standard solution. The results are expressed as mmol Trolox equivalent per g extract or 100 g seeds.

### 2.7. Ferric-Reducing Antioxidant Power (FRAP) Assay

The ferric-reducing antioxidant power (FRAP) assay was performed as previously described by Benzie and Strain [27]. The sample solution was first diluted with deionized water to fit within the linearity range. The working FRAP reagent was prepared by mixing 10 volumes of 300 mM acetate buffer, pH 3.6, with 1 volume of 10 mM TPTZ in 40 mM HCL, and with 1 volume of 20 mM FeCl_3_ × 6H_2_O. A volume of 2.25 mL of a working FRAP reagent was warmed to 37 °C. Then, 75 μL of the sample and 225 μL of deionized water were added to the FRAP reagent and the absorbance was measured at 593 nm against a reagent blank after 30 min incubation. The FRAP values were calculated and are expressed as mmol of Fe^2+^ equivalent per g extract or 100 g of seeds.

### 2.8. HPLC Analysis

Methanolic extract (20 mg) of Derek cultivar was dissolved in 2 mL of 80% methanol and filtered through a 0.45 μm cellulose acetate filter (Millipore, Warsaw, Poland). Phenolic compounds were analysed using a Shimadzu HPLC system (Shimadzu Corp., Kyoto, Japan) consisting of two LC-10AD pumps, a SCTL 10A system controller, and a SPD-M 10A photodiode array detector. The chromatography was performed using a pre-packed Luna C18 column (4 × 250 mm, 5 μm; Phenomenex, Torrance, CA, USA). Elution proceeded for 50 min in a gradient system of 5–40% acetonitrile in water adjusted to pH 2.5 with trifluoroacetic acid (TFA) [28]; the detector was set at 320, the injection volume was 20 μL, and the flow rate was 1 mL/min.

### 2.9. Statistical Analysis

The results obtained in this study are reported as the mean values of three estimates ± standard deviation. Pearson correlation was used to determine the relationship between total phenolics content, TEAC, and FRAP. Principal component analysis (PCA) and hierarchical cluster analysis (HCA) with Ward’s method using Euclidean distances were also used. Statistical and chemometric data analyses were performed using Statistica (Windows software package 8.0, Dell Inc., Tulsa, OK, USA).

## 3. Results and Discussion

### 3.1. Content of Total Phenolics Compounds

The total phenolics contents of the extracts were determined using a Folin-Ciocalteu’s phenol reagent. The results are expressed as (+)-catechin equivalents per g of the extract or 100 g seeds (Table 2). The total phenolic content ranged from 1.88 (LAT 4065/01) to 7.12 mg/g extract (LAT 4054/99) and from 20.3 (LAT 4065/01) to 70.3 mg/100 g seeds (LAT 4065/01). These concentrations are low and can be compared to those obtained previously for white bean [28] and pea [16]. Very similar total phenolic contents (20.6 and 21.3 mg/100g) were reported by Fratianni et al. [29] in two Italian varieties of grass pea, and by Wang et al. [21].in nine varieties of Canadian grass pea (16.2–37.5 mg/100 g). Higher total phenolic compounds contents in grass pea were reported by Wiszniewska and Piwowarczyk [30] and Carbonaro et al. [18]. 

### 3.2. Content of Condensed Tannins

The extracts obtained from samples 5, 10, 12, 16, 17, 21, 22, 23, and 28 contained small amounts of condensed tannins. The results expressed as absorbance at 500 nm per mg extract ranged from 0.001 to 0.004. The contents of condensed tannins reported previously for extracts of lentil, abzuki bean, faba bean, broad bean, and red bean were much higher [16].

### 3.3. Antioxidant Activity

The results of the ABTS and FRAP assays are presented in Table 2. The extracts and seeds were characterized by the Trolox equivalent antioxidant capacity (TEAC) values, ranging from 0.015 (LAT 4051/99 and LAT 4065/01) to 0.037 mmol Trolox/g extract (LAT 4054/99) and from 0.158 (LAT 4068/01) to 0.372 mmol Trolox/100 g seeds (LAT 4054/99). Ferric-reducing antioxidant power (FRAP) values varied from 0.045 (LAT 4065/01) to 0.120 mmol Fe^2+^/g extract (LAT 4054/99) and from 0.487 (LAT 4068/01) to 1.189 Fe^2+^/100 g seeds (LAT 4054/99). The results were compared to those reported previously for white bean [16,23]. Some papers reported the antioxidant capacity of grass pea seeds or their extracts determined using DPPH, ABTS, and FRAP assays, β-carotene bleaching, and H_2_O_2_ scavenge [22,31,32,33]. In general, the results were lower relative to the results reported for other leguminous seeds. For example, extracts of cow pea were characterised by TEAC and TRAP values of 0.285–0.665 TE/g extract and 0.487–1.560 mmol Fe^2+^/g extract [34].

### 3.4. HPLC Analysis

**Figure 1:**
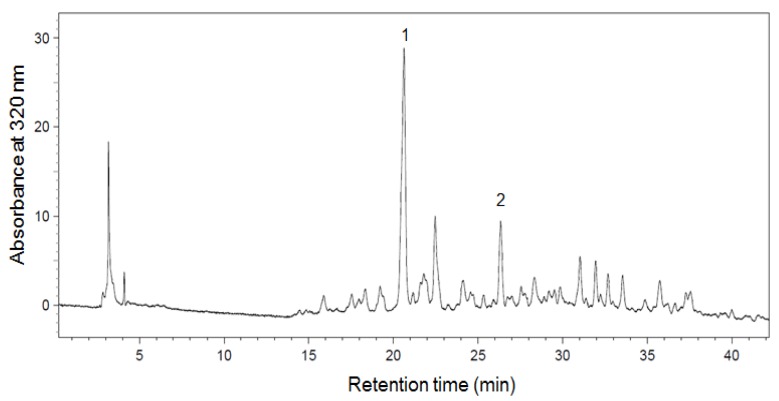
High performance liquid chromatography (HPLC) chromatogram of *Lathyrus sativus* Derek cultivar extract.

**Figure 2:**
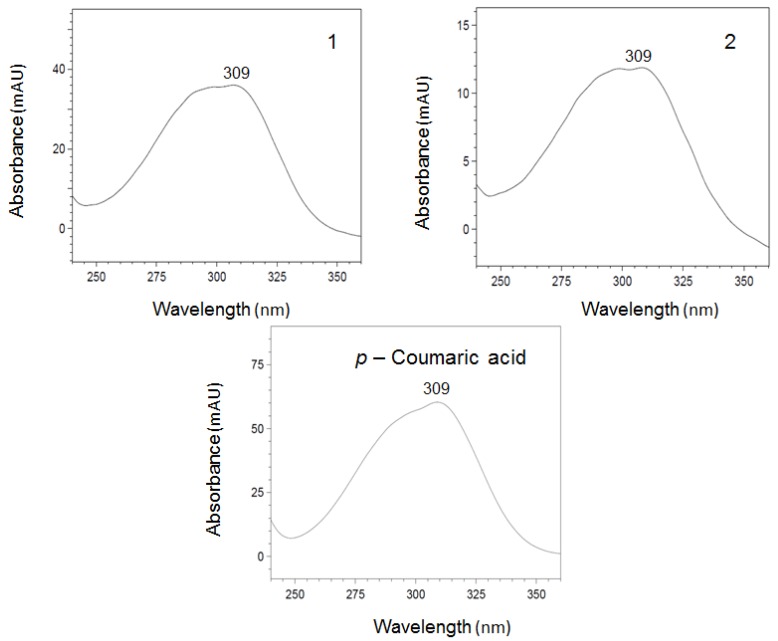
UV-diode array detector (UV-DAD) spectra of compound 1 and 2 separated using HPLC method and standard of *p-*coumaric acid.

The phenolic compounds contained in grass pea of the Derek cultivar were separated by HPLC, and the resulting chromatogram showed the presence of two major peaks (1 and 2) with retention times of 20.5 and 26.4 min, respectively (Figure 1). The UV-diode array detector (UV-DAD) spectra of compound 1 were characterized by maxima at 309 nm and were very similar to the spectrum of *p*-coumaric acid (Figure 2). The contents of compounds 1 and 2 in the extract and seeds of Derek cultivar are reported in Table 3. The presence of *p*-coumaric acid and its derivatives have been reported for lentil, broad bean, adzuki bean, and faba bean [35,36,37,38]. The high content of *p*-coumaric acid in grass pea was reported by Carbonaro et al. [18].

### 3.5. Statistical Analysis

**Figure 3:**
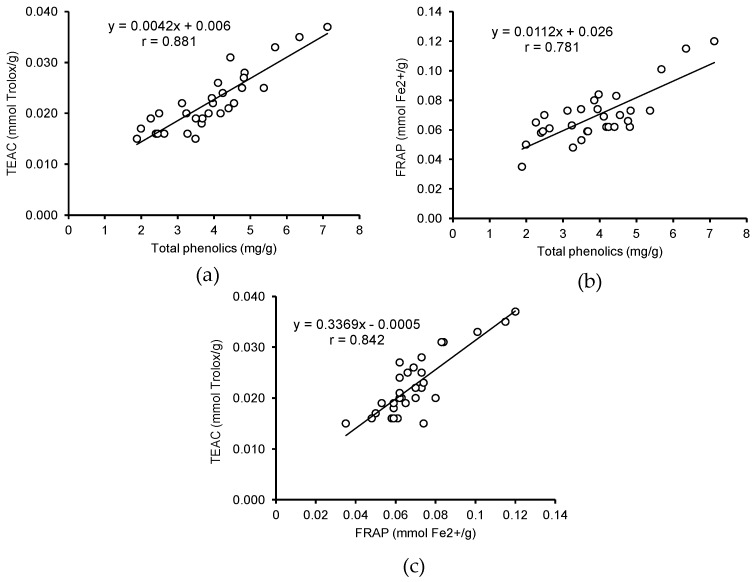
Correlation between (**a**) the total phenolics content and the results of 2,2′-azinobis-(3-ethylbenzothiazoline-6-sulfonic acid) (ABTS) assay, (**b**) total phenolics content and the results of the ferric-reducing antioxidant power (FRAP) assay, and (**c**) results of the two antioxidant assays.

In this work, for the first time, a correlation was calculated between the content of phenolic compounds in the grass pea extracts and their antioxidant activity. The correlation coefficients between the total phenolics content and the results of the ABTS and FRAP assays were 0.881 and 0.781, respectively. This correlation was also observed in the results of both assays (*r* = 0.842) (Figure 3). A similar relationship between the content of total phenolics in leguminous extracts and their antioxidant activities was previously reported by Amarowicz et al. [39] and Orac et al. [28,40].

**Figure 4:**
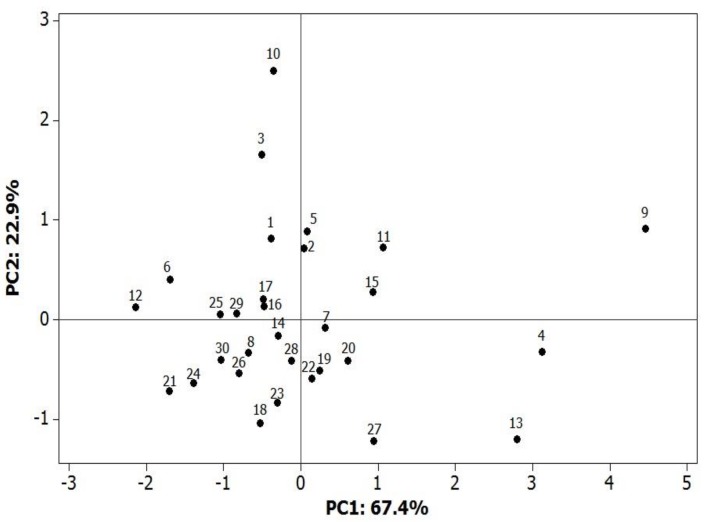
Results of the principal component analysis (PCA).

In the principal component analysis (PCA) (Figure 4), the two first components accounted for 90.3% of the total variability between the grass pea varieties. The considerable variability in terms of the analyzed traits expressed jointly with the greatest Mahalanobis distance was recorded for Italian samples 3, 4, 9, 10, and 13 (LAT 4053/99, LAT 4054/99, LAT 4064/01, LAT 4065/01, and LAT 4070/01, respectively). According to Figure 2, discrimination of the sample geographical origin by PCA was rather difficult.

**Figure 5:**
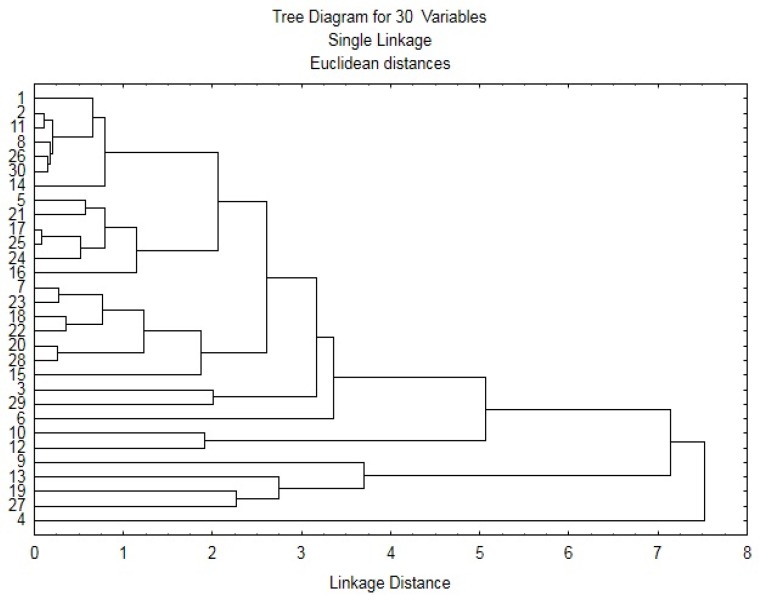
The hierarchical cluster analysis.

The hierarchical cluster analysis (Figure 5) shows several pairs of grass pea accessions (e.g., LAT 4053/99 and LAT 448; LAT 4061/01 and LAT 4006/84). Several of these pairs are, in turn, similar to each other (e.g., pair LAT 4061/01 and LAT 4006/84 and pair LAT 4063/10 and LAT 1706/92), whereas LAT 4054/99 is entirely different from all the others. The presence of similar pairs of grass peas accessions from different countries confirms the limitation of the hierarchical cluster analysis for the discrimination of the sample geographical origin.

## 4. Conclusions

Grass pea seeds with reduced content of β-ODAP after technological processing can be a source of phenolic compounds in a vegetarian or vegan diet, and in the general population. The contents of total phenolics in grass pea extract are correlated, as demonstrated by the results of the ABTS and FRAP assays. The correlation was also observed between results of both assays. Two derivatives of *p*-coumaric acid were the dominant phenolic compounds of the Derek cultivar of grass pea. In future studies, the bioavailability of grass pea phenolic compounds will be investigated in vitro.

## Figures and Tables

**Table 1:** Characteristic of grass seeds investigated in this study.

No.	Accession Code	Country of Origin	Seeds Coat Color	Weight of 100 Seeds (g)
1	LAT 4051/99	Italy	Cream to bright green	37.8
2	LAT 4052/99	Italy	Cream to bright green	42.6
3	LAT 4053/99	Italy	Cream to bright green	28.2
4	LAT 4054/99	Italy	Cream, mottled with brown edge	25.7
5	LAT 4055/99	Italy	Cream, slightly mottled and flattened	46.5
6	LAT 4056/99	Italy	Green	29.0
7	LAT 4061/99	Italy	Greyed-white with brown edge	32.1
8	LAT 4063/01	Italy	Cream-white	29.1
9	LAT 4064/01	Italy	Brick-red, dark mottled	30.0
10	LAT 4065/01	Italy	Greyed-white with brown edge	24.9
11	LAT 4068/01	Italy	Brick-red, dark mottled	29.4
12	LAT 4069/01	Italy	Cream slightly flattened	30.4
13	LAT 4070/01	Italy	Brick-red, dark mottled	24.7
14	LAT 4071/01	Italy	Cream	20.1
15	LAT 4074/01	Italy	Greyed-white with brown edge	30.0
16	LAT 4075/00	Italy	Cream with brown edge	31.3
17	LAT 4078/00	Italy	Cream to bright green with brown edge	48.0
18	LAT 4079/01	Italy	Cream with brown edge	40.9
19	LAT 4081/00	Italy	Cream	18.2
20	LAT 4082/00	Italy	Cream with brown edge	21.4
21	LAT 456/75	Spain	Cream with dark edge	28.5
22	LAT 1706/92	Spain	Cream with short black edge	27.7
23	LAT 4006/84	Spain	Cream with brown edge	23.5
24	LAT 4007/84	Spain	Cream	25.3
25	LAT 4085/73	Spain	Cream	26.1
26	LAT 444/73	Germany	Cream, brown edge, slightly mottled	16,7
27	LAT 478	Germany	Gray, dark brown edge, slightly mottled	19.5
28	LAT 447	France	Cream, slightly brick-red	16.4
29	LAT 448	France	Cream	17.9
30	Cultivar Derek	Poland	Bridge-cream	11.8

LAT means “*Lathyrus*”. Seed size (100 seeds): below 15 g: small; 15–25 g: medium, and above 25 g: large.

**Table 2:** Characteristics of the grass pea seeds and their extracts: content of total phenolics and antioxidant activity.

No.	Total Phenolics ^1^		ABTS ^2^ Assay		FRAP ^3^ Assay	
	mg/g Extract	mg/100 g Seeds	mmol TE/g Extract	mmol TE/100 g Seeds	mmol Fe^2+^/g Extract	mmol Fe^2+^/100 g Seeds
1	3.49 ± 0.07	40.6 ± 0.8	0.015 ± 0.000	0.170 ± 0.001	0.074 ± 0.001	0.859 ± 0.009
2	3.85 ± 0.04	41.6 ± 0.4	0.020 ± 0.000	0.214 ± 0.001	0.080 ± 0.002	0.861 ± 0.021
3	2.63 ± 0.03	30.5 ± 0.4	0.016 ± 0.000	0.186 ± 0.003	0.061 ± 0.001	0.713 ± 0.017
4	7.12 ± 0.10	73.0 ± 1.1	0.037 ± 0.001	0.372 ± 0.014	0.120 ± 0.001	1.028 ± 0.012
5	3.12 ± 0.04	36.1 ± 0.4	0.022 ± 0.000	0.253 ± 0.001	0.073 ± 0.002	0.851 ± 0.021
6	2.40 ± 0.03	27.2 ± 0.4	0.016 ± 0.000	0.176 ± 0.001	0.058 ± 0.001	0.654 ± 0.010
7	3.66 ± 0.04	48.2 ± 0.5	0.018 ± 0.000	0.241 ± 0.003	0.059 ± 0.001	0.780 ± 0.015
8	3.68 ± 0.02	41.6 ± 0.2	0.019 ± 0.000	0.210 ± 0.004	0.059 ± 0.001	0.662 ± 0.004
9	6.35 ± 0.10	65.5 ± 1.0	0.031 ± 0.001	0.319 ± 0.0013	0.115 ± 0.001	1.189 ± 0.008
10	1.88 ± 0.07	20.3 ± 0.8	0.015 ± 0.000	0.232 ± 0.002	0.045 ± 0.003	0.487 ± 0.002
11	3.97 ± 0.17	41.6 ± 1.8	0.022 ± 0.000	0.158 ± 0.002	0.084 ± 0.002	0.889 ± 002
12	1.99 ± 0.05	22.1 ± 0.6	0.017 ± 0.000	0.191 ± 0.004	0.101 ± 0.002	0.557 ± 0.008
13	5.68 ± 0.10	61.8 ± 1.1	0.033 ± 0.001	0.229 ± 0.002	0.069 ± 0.002	1.105 ± 0.002
14	4.11 ± 0.09	42.4 ± 0.9	0.026 ± 0.001	0.232 ± 0.002	0.069 ± 0.001	0.707 ± 0.024
15	4.45 ± 0.07	45.0 ± 0.7	0.031 ± 0.001	0.309 ± 0.002	0.083 ± 0.002	0.835 ± 0.018
16	3.94 ± 0.03	38.6 ± 0.3	0.023 ± 0.000	0.225 ± 0.002	0.074 ± 0.001	0.724 ± 0.006
17	3.24 ± 0.03	37.4 ± 0.4	0.020 ± 0.000	0.229 ± 0.001	0.063 ± 0.002	0.728 ± 0.021
18	4.18 ± 0.07	47.2 ± 0.7	0.020 ± 0.000	0.227 ± 0.001	0.062 ± 0.002	0.698 ± 0.021
19	5.37 ± 0.07	56.8 ± 0.7	0.025 ± 0.001	0.263 ± 0.004	0.073 ± 0.001	0.769 ± 0.006
20	4.84 ± 0.03	49.5 ± 0.7	0.028 ± 0.001	0.284 ± 0.006	0.073 ± 0.001	0.740 ± 0.012
21	3.27 ± 0.04	35.7 ± 0.4	0.016 ± 0.000	0.179 ± 0.001	0.060 ± 0.003	0.524 ± 0.030
22	4.24 ± 0.07	46.9 ± 0.8	0.024 ± 0.001	0.269 ± 0.001	0.062 ± 0.001	0.678 ± 0.09
23	4.55 ± 0.07	48.0 ± 0.3	0.022 ± 0.000	0.230 ± 0.004	0.060 ± 0.001	0.655 ± 0.010
24	3.50 ± 0.04	36.9 ± 0.3	0.019 ± 0.000	0.196 ± 0.003	0.053 ± 0.002	0.557 ± 0.018
25	2.45 ± 0.03	37.4 ± 0.5	0.016 ± 0.000	0.180 ± 0.003	0.059 ± 0.002	0.687 ± 0.030
26	4.40 ± 0.06	41.4 ± 0.7	0.021 ± 0.000	0.212 ± 0.004	0.062 ± 0.002	0.622 ± 0.024
27	4.82 ± 0.10	59.1 ± 0.7	0.027 ± 0.001	0.293 ± 0.006	0.062 ± 0.003	0.683 ± 0.028
28	2.49 ± 0.07	49.7 ± 0.3	0.020 ± 0.000	0.205 ± 0.007	0.070 ± 0.003	0.723 ± 0.031
29	4.77 ± 0.11	32.5 ± 0.4	0.025 ± 0.001	0.244 ± 0.004	0.066 ± 0.001	0.659 ± 0.010
30	2.26 ± 0.02	41.3 ± 0.7	0.019 ± 0.000	0.180 ± 0.001	0.065 ± 0.001	0.638 ± 0.007

^1^ As (+)-catechin equivalents; ^2^ 2,2′-azinobis-(3-ethylbenzothiazoline-6-sulfonic acid); ^3^ Ferric-Reducing Antioxidant Power.

**Table 3:** Content of two main phenolic compounds in Derek cultivar of grass pea.

Phenolic Compound	Content ^1^	
	mg/g Extract	mg/g 100 g Seeds
1	1.15 ± 0.05	20.7 ± 2.7
2	0.48 ± 0.03	8.64 ± 0.54

^1^*p-*coumaric acid equivalents.
